# Characteristics of frequent emergency department presenters to an Australian emergency medicine network

**DOI:** 10.1186/1471-227X-11-21

**Published:** 2011-12-16

**Authors:** Donna Markham, Andis Graudins

**Affiliations:** 1 Monash Medical Centre Emergency Department Care Co-ordination Team and Allied Health, Southern Health, Melbourne, Australia; 2 Department of Medicine, Southern Clinical School, Faculty of Medicine, Nursing and Health Sciences, Monash University, Clayton, Victoria, Australia; 3 Southern Health Emergency Medicine Program, Department of Emergency Medicine, Monash Medical Centre, Clayton, Victoria, Australia

## Abstract

**Background:**

To describe the characteristics of emergency department (ED) patients defined as frequent presenters (FP) presenting to an Australian emergency department network and compare these with a cohort of non-frequent presenters (NFP).

**Method:**

A retrospective chart review utilising an electronic emergency medicine patient medical record database was performed on patients presenting to Southern Health EDs from March 2009 to March 2010. Non-frequent presenters were defined as patients presenting less than 5 times and frequent presenters as presenting 8 or more times in the study period. Characteristics of both groups were described and compared.

**Results:**

During the 12-month study period there were 540 FP patients with 4549 admissions and 73,089 NFP patients with 100,943 admissions. FP patients were slightly older with a significant increase in frequency of patients between the ages of 70 to 79 years and they were more likely to be divorced or separated than NFP patients. Frequent presenters to the emergency department were more likely to utilise the ambulance service to arrive at the hospital, or in the custody of police than NFP patients. FPs were more likely to be admitted to hospital, more likely to have an admission to a mental health bed than NFP patients and more likely to self-discharge from the emergency department while waiting for care.

**Conclusions:**

There are major implications for the utilisation of limited ED resources by frequent presenters. By further understanding the characteristics of FP we may be able to address the specific health care needs of this population in more efficient and cost effective ways. Further research analysing the effectiveness of targeted multidisciplinary interventions aiming to reduce the frequency of ED attendances may be warranted.

## Background

The increasing demand placed on hospital Emergency departments (EDs) by patients who frequently present has been well documented in studies from North America and the United Kingdom [1-9]. However, there is a paucity of Australasian literature describing the characteristics of this patient group and further definition is required. The ED is often utilised by patients with complex health care needs including those with multiple medical co-morbidities, and long-standing social, behavioural and psychological care requirements. This group may place a large demand on pre-hospital and emergency department resources and individuals often present on multiple occasions each year [10,11]. Frequent presenters have been reported to contribute to between 1.4- 4% of total ED attendances [6,7,12,13].

There are many common assumptions made about this group, and research definitions of frequent presenters vary. Previous studies of frequent ED presenters have indicated that this is not a homogeneous group and may have a multitude of reasons for presenting to hospital. These may include ongoing management of chronic illnesses, psychological stressors associated with physical illness, social problems related to their medical problems and other issues that may not be directly addressed by the acute medical services provided by EDs [8,10,11]. Given the episodic nature of care provided in the ED, patients may undergo over or under investigation of their acute problem and there may be a failure to appreciate the underlying cause for their presentation [14]. These factors have major implications for the utilisation of limited acute health care resources by this small group of patients that may be managed in more cost effective ways.

Previous studies have varyingly defined frequent ED presenters as those with between 4 and 10 admissions per year [11,14,15].

To date, there have been three known Australian studies examining frequent ED presenters. Jelinek et al (2008), described the changing characteristics of frequent presenters depending on the frequency of attendances to urban EDs in Western Australia. This study reported that most FPs were presenting fewer than 20 times per year and had more serious and urgent illnesses than other patients, more often requiring in-patient services [11]. Wooden et al (2009) looked specifically at frequent presenters with mental disorders and assessed the care those patients received in ED. This study reported this patient group comprised 4.5% of total ED attendances and documented management appeared to be less than optimal [16] (2003) focused on the suitability of these patients for diversion to general practice and concluded that the 'majority of the heaviest users of an ED are not suitable for general practice', and attempting diversion may not be successful [17]. Our study aims to further define characteristics for all frequent presenters groups and address the paucity of research in the Australian health care setting.

Furthermore, studies describing frequent ED presenters in the UK and USA may not be directly relevant to the Australasian health care model. Both countries have different health care and insurance systems as well as varying ED patient populations to Australia [6-8,18]. As a result, this study was undertaken to better define the characteristics of frequent ED presenters to a public health service in Victoria, Australia.

## Method

The Southern Health hospital network, services a population of 888,163 people or 22% of metropolitan Melbourne, via three EDs with over 165,000 attendances annually.

This was a retrospective case-control study comparing two patient populations: frequent ED presenters (FP) and non-frequent ED presenters (NFP). Ethics approval was obtained from the Southern Health Human Research and Ethics Committee.

A literature review was initially conducted using Cinahl, Cochrane Medline, Proquest, Publine and Google Scholar, to identify previous reports that examined frequent ED presenters. Search terms used included: frequent presenters, frequent flyers, frequent visitors, frequent attenders, re-presenters, readmissions and emergency department. Based on this review, FPs were defined as those patients having 8 or more attendances in a 12 month period and NFPs as those with 5 or less. Eight was chosen arbitrarily as the descriptor of FPs as it was in the mid-range (median value 6, range 3-20) of previous descriptive studies [1,4,8,9,11-13,18].

Data on ED attendances were collected using the Symphony, Electronic Patient Records and Medical Record database (Ascribe Symphony, United Kingdom) used in all Southern Health EDs. Electronic abstraction methods were used and the electronic data were interrogated based on search terms. The abstractor was an ED physician with no association with the study however had previous experience and training with extracting data from the Symphony program. The authors did not test for inter-rater agreement. All adult patient attendances from March 2009 - March 2010 were extracted. Information obtained included age, sex, marital status, triage date, triage category, type of accompanying person, arrival mode, presenting complaint, discharge diagnosis, disposition, length of stay in ED, usual residence, primary language, allied health intervention, and country of birth. The data were then entered into a Microsoft Excel spreadsheet for further analysis. This group was comprised of 3767 attendances during the study period. Patients' ages ranged from 19 to 105 years.

### Exclusion criteria

Adults who had 6 or 7 attendances and children up to and including the age of 18 years.

Diagnoses were categorised into 12 subgroups according to VEMD (Victorian Emergency Minimum Dataset) diagnosis codes supplied by Victorian Department of Human Services on patient discharge from the ED.

Descriptive data were expressed as medians with interquartile range or as number of cases with percentages as appropriate. Median values are reported given the propensity for non-normal distribution of data, particularly seen with variables such as age and length of stay. Univariate comparisons of specific characteristics of the two patient groups were made using Chi squared analysis for categorical variables with report of odds ratios and 95% Confidence Intervals (CI). Continuous variables were analysed using the unpaired t-test with Welch's correction applied to non-normally distributed data. Statistical significance was defined as a p < 0.05. Statistical analysis was performed using GraphPad InStat Version 3.0 (GraphPad Software Inc, La Jolla, CA, USA).

## Results

During the 12-month study period there were 540 frequent presenter (FP) patients with 4549 admissions (median number admission per patient = 10 (IQ range 8-12)) and 73,089 non-frequent presenter (NFP) patients with 100,943 admissions (median = 1 (IQ range 1-2)). There were a total of 109,259 adult presentations to the EDs in the study period with the inclusion of the patients with 6 and 7 presentations. As a result, FP patients were responsible for 4.2% of all adult ED presentations.

Demographic data are summarised in Table 1. FP patients were slightly older with a significant increase in frequency of patients between the ages of 70 to 79 years (FP 14.4% v NFP 9.7%, OR1.6, 95% CI: 1.2 to 2.0, p = 0.0003). Frequent presenters were also more likely to be divorced or separated than NFP patients (13.6% v 6.5%, OR 2.2, 95% CI 1.7-2.8, p < 0.0001).

**Table 1:** Demographic and marital status data comparing frequent presenters (FP) and non-frequent presenters (NFP)

	FP v NFP (n = 540 v n = 73089)	OR	95% CI	p-value
GENDER	51.6%M v 48.2%M	1.1	0.96 to 1.4	NS

AGE Median (IQ range)	47 yrs (33-68) vs 45 yrs (30-64)	-		0.02

Age 19-29 yrs	18.9% v 23.4%	0.8	0.62 to 0.95	0.02

Age 70-79 yrs	14.4% v 9.7%	1.6	1.2 to 2.0	0.0003

				

Married/defacto	41.8% v 53.9%	0.61	0.52 to 0.73	< 0.0001

Divorced/separated	13.6% v 6.5%	2.2	1.7 to 2.8	< 0.0001

Single	29.1% v 29.1%	1.0	0.83 to 1.2	NS

Widowed	8.9% v 7.3%	1.2	0.91 to 1.6	NS

Odds ratio (OR) and Confidence Interval (CI) are used.

Frequent presenters to the emergency department were more than twice as likely to utilise the ambulance service to arrive at the hospital than NFP patients (51% v 31%, OR 2.4, 95%CI: 2.3-2.6, p < 0.0001). There was no increased acuity in FP patients when assessed by their Australasian triage score on presentation for each group. A comparison of frequency of triage category assessment between groups did not show any differences other than a slightly higher number of Australasian Triage category 3 patients in the FP group. Frequent presenters were three times as likely to present to the emergency department in the custody of the police (1.7% v 0.6%, OR 3.1, 95%CI: 2.4-3.9, p < 0.0001). The ED length of stay for FP group v NFP was not significant.

Analysis of the disposition of frequent presenters showed that this group of patients were more likely to be admitted to hospital (29% v 26.3%, OR 1.1, 95%CI: 1.07-1.2, p < 0.0001), more likely to have an admission to a mental health bed than NFP patients (2.9% v 0.9%, OR 3.3, 95%CI: 2.7-3.9, p < 0.0001) and more likely to self-discharge from the emergency department while waiting for care than NFP patients (10.1% v 5.9%, OR = 1.7, 95%CI: 1.6-1.9, p < 0.0001).

**Figure 1:**
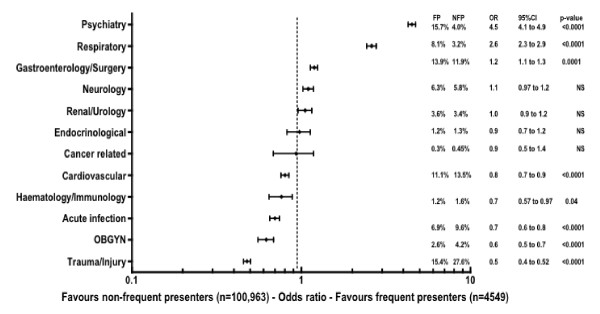
**Forest plot comparing admission diagnoses for frequent presenters and non-frequent ED presenters**.

Comparison of admission diagnoses of FP and NFP groups revealed that frequent presenter patients were more likely to have an emergency department discharge diagnosis of a psychiatric problem (15.7% v 4.0%, OR 4.5, 95%CI: 4.1-4.9, p < 0.0001) or a respiratory complaint (8.1% v 3.2%, OR 2.6, 95%CI: 2.3-2.9, p < 0.0001). These two groups combined comprised 24% of all admission diagnoses from the emergency department for FP patients. NFP patients were more likely to have a diagnosis related to acute infective (6.9% v 9.6%, OR 0.7, 95%CI: 0.6-0.8, p < 0.0001), trauma-related (15.4% v 27.6%, OR 0.5, 95%CI: 0.4-0.52, p < 0.0001) or gynaecological problem (2.6% v 4.2%, OR 0.6, 95%CI: 0.5-0.7, p < 0.0001). These three diagnosis categories comprised 41% of all NFP presentation diagnoses. Emergency department diagnosis data are summarised in Figure 1.

## Discussion

This study has assisted in better identifying a number of patient populations who may benefit from a targeted multidisciplinary approach in an Emergency Department setting. Such an approach must address the complex health needs of this vulnerable population. Frequent presenters represented 0.7% of adult ED patients, in our population, and 4.2% of all adult ED presentations during the 12-month study period. These data are consistent with observations from studies undertaken overseas [1,7,12,13]. In particular, we observed that one-quarter of FPs presented with either a psychiatric or respiratory complaint, suggesting that these two diagnosis groups may be the focus of particular interventions to reduce re-attendance. In addition to these diagnostic patient groups the study identified psychosocial factors that should be addressed in any approach for this vulnerable population.

There are many negative associations with FPs who are often labelled as 'frequent flyers'. The complexities of their health care needs may be overlooked due to various misconceptions. Frequent Presenters may be perceived as time consuming, illegitimate users of the ED, leading to the development of staff indifference towards these patients [10]. There may be a tendency to divert frequent ED presenters to general practice to address their complex health care needs. However, previous epidemiologic studies suggest that these patients are not general practice patients, and that simple diversion to primary health care is not the answer in many cases [15,17]. In fact, frequent ED presenters may be better cared for when they attend an ED that is supported by a multidisciplinary team providing medical, nursing, allied health and mental health assessment in a collaborative and timely way. This includes liaison with GPs, ambulance services, case managers, family members and other community care providers.

Frequent presenters not only have a significant impact on the use of ED resources but also may have an impact on the utilisation of pre-hospital resources. This is evidenced by the large percentage of FPs that arrived via ambulance in our study. Interestingly, Ambulance Victoria has developed a referral service for patients who are frequent callers for transport to hospital in an attempt to reduce unnecessary utilisation of acute care ambulances for patient transport (*Ambulance Victoria, Referral Service*).

Emergency department case management of FPs has been reported to increase attendances in some studies [15]. However, these studies excluded large FP populations who already received case management support. They demonstrated that multidisciplinary case management has been shown to have a positive effect on psychosocial factors for FPs. Similarly, individual care plans for specific patient groups reduce hospital admissions and decrease the number of investigations carried out in selected patients [19]. In addition to ED care plans, targeted interventions may also be effective in reducing FPs [9,10,13,20,21]. Development of care plans to address gaps in service delivery may be warranted. These particularly include understanding the complex psychosocial needs of chronic psychiatric and respiratory frequent presenters that are frequently neglected despite multiple ED visits. Under the supervision of ED Care Co-ordination/Allied Health Teams, the Frequent Presenters Program at Southern Health, has instituted and monitors the ED care plans of patients who frequently present to the ED with complex and co-existing medical, social, behavioural and psychological needs. The plans include close liaison with community-based medical and allied health teams, as well as hospital-based outreach programs to supplement community care in times of acute stress for individual patients. Future research aims to assess the effectiveness of these strategies in the target populations.

## Limitations

This study aimed to identify patients by frequency of presentation to the ED. As a result, we only analysed the ED admission electronic medical record data-base. We did not review hospital records of admitted patients. We did not look at the hospital LOS or outcome of the admitted patients. Diagnoses were those made at end of ED stay and not hospital discharge diagnoses for admitted patients.

The retrospective nature of the study also limited the ability to collect additional information on other potential differences between the two groups.

Finally, this study only explores frequent presenters in one health care network and does not make comparisons with data from other metropolitan hospitals in varying health care settings.

## Conclusions

We report an analysis of characteristics of frequent emergency department presenters in an Australasian setting. Frequent Presenters in our hospital network had significant mental health and chronic respiratory health problems relying heavily on ambulance and acute care resources. These observations suggest that a potential gap may exist in community and home care services in supporting these patient groups. Emergency department care co-ordination teams have the potential to identify frequent presenter patients and facilitate the development of targeted care plans for specific patients. These should include close liaison with community allied health and medical services to reduce unnecessary re-presentation to hospital.

## Competing interests

The authors declare that they have no competing interests.

## Authors' contributions

DM conceived the study and drafted the manuscript. AG assisted in the design of the study, performed the statistical analysis and contributed to the manuscript. Both authors have read and approved the final manuscript

## Pre-publication history

The pre-publication history for this paper can be accessed here:

http://www.biomedcentral.com/1471-227X/11/21/prepub

## References

[B1] Mandelberg JH Kuhn RE Kohn MA Epidemiologic analysis of an urban, public emergency departments frequent users Academic emergency medicine 2000 7 6 637 646 10.1111/j.1553-2712.2000.tb02037.x 10905642

[B2] Robertson C McConville P Lefevre P Psychiatric characteristics of frequent attenders at accident and emergency: a case register comparison with non frequent attenders SMJ 2005 50 2 75 76 10.1177/003693300505000211 15977520

[B3] Bentley J Meyer J Repeat attendance by older people at accident and emergency departments Journal of Advanced Nursing 2004 48 2 149 156 10.1111/j.1365-2648.2004.03182.x 15369495

[B4] Byrne M Murphy AW Plunkett PK McGee HM Murray A Bury G Frequent attenders to an emergency department: A study of primary health care use, medical profile, and psychosocial characteristics Annals of Emergency Medicine 2003 41 3 309 318 10.1067/mem.2003.68 12605196

[B5] Michelen W Martinez J Lee A Wheeler DP Reducing Frequent Flyer Emergency Department Visits Journal of Health Care for the Poor and Undeserved 2006 17 59 69 10.1353/hpu.2006.0010 16520511

[B6] Sandoval E Smith S Walter J Henning Schuman SA Olson MP Striefler R Brown S Hickner J A comparison of frequent and infrequent visitors to an urban emergency department The Journal of Emergency Medicine 2010 38 2 115 121 10.1016/j.jemermed.2007.09.042 18462906

[B7] LaCalle E Rabin E Frequent users of Emergency Departments: The Myths, the Data and the Policy Implications Annals of Emergency Medicine 2010 56 1 42 48 10.1016/j.annemergmed.2010.01.032 20346540

[B8] Moore L Deehan A Seed P Characteristics of frequent attenders in an emergency department: analysis of 1-year attendance data EMJ 2009 26 263 267 19307386 10.1136/emj.2008.059428

[B9] Cook LJ Knight S Junkins EP Mann NC Dean JM Olson LM Repeat patients to the emergency department in a statewide database Academic Emergency Medicine 2004 11 3 256 263 10.1111/j.1553-2712.2004.tb02206.x 15001405

[B10] Fulde GWO Duffy M Emergency Department frequent flyers: unnecessary load or lifeline? MJA 2006 184 12 595 16803432 10.5694/j.1326-5377.2006.tb00407.x

[B11] Jelinek JA Jiwa M Gibson NP Lynch AM Frequent attenders at emergency departments: a linked-data population study of adult patients MJA 2008 189 10 552 556 19012551 10.5694/j.1326-5377.2008.tb02177.x

[B12] Kennedy D Ardagh M Frequent attenders at Christchurch Hospitals Emergency Department: a 4 -year study of attendance patterns The New Zealand Medical Journal 2004 117 1193 871 878 15133521

[B13] Helliwell PE Hider PN Ardagh MW Frequent attenders at Christchurch Hospitals emergency department The New Zealand Medical Journal 2001 114 160 161 11400923

[B14] Skinner J Carter L Haxton C Case management of patients who frequently present to a Scottish emergency department Emergency Medicine Journal 2009 26 2 103 105 10.1136/emj.2008.063081 19164618

[B15] Phillips GA Brophy DS Weiland TJ Chenhall AJ Dent AW The effect of multidisiplinary case management on selected outcomes for frequent attenders at an emergency department MJA 2006 184 12 602 606 16803437 10.5694/j.1326-5377.2006.tb00412.x

[B16] Wooden MDG Air TM Schrader GD Wieland B Goldney RD Frequent attenders with mental disorders at a general emergency department Emergency Medicine Australasia 2009 21 3 191 195 10.1111/j.1742-6723.2009.01181.x 19527278

[B17] Dent AW Phillips GA Chenhall AJ McGreggor LR The heaviest repeat users of an inner city emergency department are not general practice patients Emergency Medicine 2003 15 322 329 10.1046/j.1442-2026.2003.00470.x 14631698

[B18] Locker TE Baston S Mason SM Defining frequent use of an urban emergency department EMJ 2007 24 398 401 17513534 10.1136/emj.2006.043844 PMC2658272

[B19] Newton A Sarker SJ Parfitt A Individual care plans can reduce hospital admission rate for patients who frequently attend the emergency department EMJ 2010 published online 10.1136/emj.2009.085704 20515901

[B20] Moore G Gerdtz M Manias E Hepworth G Dent A Socio-demographic and clinical characterisitcs of re-presentiaon to an Australian inner-city emergency department: implications for service delivery BMC Public Health 2007 7 320 329 10.1186/1471-2458-7-320 17996112 PMC2222161

[B21] Cardin S Afilalo M Lang E Collet JP Colacone A Tselios C Dankoff J Guttman A Intervention to decrease Emergency Department crowding: does it have an effect on return visits and hospital readmissions? Annals of Emergency Medicine 2003 41 2 173 185 10.1067/mem.2003.50 12548266

